# Non-Coding RNAs and Human Diseases: Current Status and Future Perspectives

**DOI:** 10.3390/ijms241411679

**Published:** 2023-07-20

**Authors:** Justyna Niderla-Bielińska, Ewa Jankowska-Steifer, Paweł Włodarski

**Affiliations:** 1 Histology and Embryology Department, Medical University of Warsaw, 02-097 Warsaw, Poland; ewa.jankowska-steifer@wum.edu.pl; 2 Department of Methodology, Center for Preclinical Research, Medical University of Warsaw, 1B Banacha Street, 02-097 Warsaw, Poland; pawel.wlodarski@wum.edu.pl

Non-coding RNAs (ncRNAs) are a family of RNA molecules that, unlike messenger RNAs, are not templates for protein synthesis but have an essential or regulatory role in this process. As they are the key elements of the ribosomes (rRNA) or transferring amino acids (tRNA) to the assembled peptide, ncRNAs are critical for translation. What became known only 30 years ago was that other ncRNAs are also involved in the regulation of protein expression or other biochemical processes [1]. These latter ones, often referred to as the regulatory ncRNA, immediately gained the interest, which continues to grow today, since new evidence is demonstrated almost every day that proves their involvement in processes such as cell proliferation, apoptosis, and differentiation [2]. According to the number of nucleotides, cytoplasmic regulatory ncRNAs are divided into two classes—microRNAs (miRNA) and long non-coding RNAs (lncRNAs) [3]. miRNAs interact in a sequence-specific manner with mRNA transcripts through the recognition of complementary sites at the UTR region of the mRNA, causing transcriptional repression or its degradation [4]. RNA molecules longer than 200 nucleotides are defined as lncRNAs. These particles regulate expression mostly via the interactions with the regulatory elements of transcription. lncRNAs may positively or negatively affect enhancer activity either by forming chromatin loops with target genes or competing with the enhancer, respectively. Moreover, lncRNAs have been shown to interact with the transcript and to inhibit splicing [3]. Interestingly, lncRNAs can also attract and adsorb miRNAs, which reduces the activity of miRNAs. Such stratified interactions of different types of RNA illustrate the complex interplay of these regulatory ncRNAs [3].

ncRNAs impact gene expression exerting countless effects on various cellular mechanisms [5]. Dysbalanced expression of ncRNAs may cause numerous human disorders, such as cancer, metabolic syndrome, cardiovascular, autoimmune or infectious diseases [6,7,8,9]. Non-coding regulatory transcripts can be released into the extracellular space and bloodstream within the exosomes which makes them highly stable and resistant to enzymatic degradation. Thus, ncRNAs can serve as diagnostic or prognostic biomarkers [10]. Finally, regulatory ncRNAs are considered therapeutic agents for the treatment of various diseases [11]. ncRNA-based gene silencing that targets and suppresses genes associated with specific pathologies, including cancer, led to the development of a new class of therapeutics, some of which are FDA approved [12]. It is crucial to understand the mechanisms of interaction between regulatory ncRNAs and their targets to design the most effective diagnostic tools and treatment modalities.

This Special Issue of the *International Journal of Molecular Sciences*, entitled “Non-coding RNAs and Human Diseases: Current Status and Future Perspectives” includes the latest studies and reviews on the mechanisms of ncRNA function and its potential use in medicine. The current Special Issue includes seven articles that focus on the function of ncRNAs and their application in diagnostics or therapy.

In “Circulating MicroRNA Profiling Identifies Distinct MicroRNA Signatures in Acute Ischemic Stroke and Transient Ischemic Attack Patients”, using RNA-seq, Toor et al. identify 11 miRNAs that are differentially expressed and present in serum samples collected from patients suffering either from transient ischemic attack (TIA) or acute ischemic stroke (AIS). The authors developed a multivariate classifier with potential utilization as a discriminative biomarker for AIS and TIA patients. Understanding the differences in molecular pathways in AIS and TIA has merit for deciphering the underlying cause for neuronal deficits with long-term effects and high risks of morbidity and mortality [13].

In “Non-Coding RNAs in Tuberculosis Epidemiology: Platforms and Approaches for Investigating the Genome’s Dark Matter”, Almatroudi reviews ncRNAs as potential therapeutic targets in the treatment of tuberculosis (TB). He also focuses on current platforms, experimental strategies, and computational analyses to explore ncRNAs in TB. In the article, cutting-edge data are presented on the key challenges and novel techniques for the future and for a wide-range of therapeutic applications of ncRNAs [14].

In “Advances of Epigenetic Biomarkers and Epigenome Editing for Early Diagnosis in Breast Cancer”, Sarvari et al. focus on the ncRNAs in breast cancer progression, diagnosis, and treatment. The authors defend the statement that future medical treatment for breast cancer patients will likely depend upon a better understanding of epigenetic modifications. The review aims to outline different epigenetic mechanisms including DNA methylation, histone modifications, and ncRNAs with their impact on breast cancer [15].

In “The Roles of miRNAs in Predicting Bladder Cancer Recurrence and Resistance to Treatment”, Das et al. review the importance of miRNAs as molecular biomarkers in determining the likelihood of recurrence and response to treatment of bladder cancer (BC). The recurrence of BC presents a significant issue and is managed in the clinical setting with intravesical chemotherapy or immunotherapy. miRNAs can be used not only as predictors of recurrence, but also as markers for resistance or susceptibility to BC treatment [16].

In “Using ncRNAs as Tools in Cancer Diagnosis and Treatment—The Way towards Personalized Medicine to Improve Patients’ Health”, Piergentili et al. summarize the present knowledge regarding ncRNA functions, interactions, and impact on both health and disease. The review illustrates how ncRNAs can be used for disease treatment, the current challenges and pitfalls, and the roles of environmental and lifestyle-related contributing factors, in addition to the ethical, legal, and social issues arising from their improper use [17].

In “Methylation-Regulated Long Non-Coding RNA Expression in Ulcerative Colitis”, Fenton et al. highlight the role of epigenetic processes such as DNA methylation and lncRNA expression in ulcerative colitis (UC). The authors evaluated lncRNA expression in UC and healthy patients, which may be affected by epigenetic changes, and found several molecules associated with the regulation of genes involved in inflammatory immune response in UC. The obtained data, which unravel the interplay between lncRNA expression regulated by DNA methylation in UC, might improve the understanding of UC pathogenesis [18].

Finally, in “miR-31-5p-Modified RAW 264.7 Macrophages Affect Profibrotic Phenotype of Lymphatic Endothelial Cells In Vitro”, Moskalik et al. evaluate the influence of macrophages modified by miR-31-5p, a molecule that regulates fibrosis and lymphangiogenesis, on lymphatic endothelial cells (LECs) in vitro. Myocardial fibrosis and structural changes in cardiac lymphatic vessels in metabolic syndrome contribute to the severity of heart failure. Thus, the mechanisms leading to the development of the above processes are crucial for understanding the pathophysiology of this condition. The obtained results suggest that macrophages under the influence of miR-31-5p show the potential to inhibit LEC-dependent fibrosis; however, more studies are needed to confirm this effect in vivo [19].

In summary, this Special Issue of the *International Journal of Molecular Sciences* presents a collection of articles that deliberate the latest data on ncRNAs, emphasizing their utility as a diagnostic or therapeutic tool in human diseases.
